# Supplementary material for: Learning curve and surgical outcome of robotic assisted colorectal surgery with ERAS program
Source: Sci Rep. 2022 Nov 29;12:20566. doi: 10.1038/s41598-022-24665-w (PMC9709162; doi:10.1038/s41598-022-24665-w)
Supplement: Supplementary file 1 — Supplementary Information 1. [file 41598_2022_24665_MOESM1_ESM.zip › LSC Raw data-Ñ[▒K/135/O2_ñw╝╨░O▒Kñσ.pdf]

正式摘要(電子簽章：000000223723716)

請選擇...reporter

臺中榮民總醫院

Operation Note

[redacted]

- 手術日期：2021/08/27
- 手術醫師：劉嘯天，賴家鈺，R 蔡濬樟
- Anesthesia：GE
- Position：Supine
- Aseptic agents：Soap，Betadine Alcohol

Diagnosis

- Pre-operative diagnosis : Liver mets, S6.
- Post-operative diagnosis : - Liver tumors, suspect MCRC, S6, deep S5.

  - Liver cyst, S1, S8.

Surgical Indication

- - Metastasectomy,
  - ICGR15=4.88%, T1/2=3.4mins.

Operation

- - Lap. S8, S6 subsegment hepatectomies.

Finding

- - Finding:
  = No liver cirrhosis,
  = Visible tumor over S6.
  = Invisible tumor within S5,
  = No gallbladder stone.
  - Method:
  = Hepatectomy time between 15:40 and 18:42.
  = Pringle 9 times, 15min35sec, 15min45sec, 16min00sec, 15min33sec, 14min55sec, 11min43sec, 16min46sec, 09min28sec, 22min36sec.
  - Specimen:
  = Total specimen weight 80 gram.
  = Liver S5, tumor 1x1cm, margin 1mm.
  = Liver S6, tumor 3x1.5cm, no surgical margin.
  - EBL: 1275ml.
  - Transfusion: 4u FFP.

Procedure

- Under general anesthesia, patient was put into lithitomy position. CRS doctor performed laparoscopic LAR first. I took over for further liver surgery. Six working poles were made. Liver was moblized by dissection of right triangular and falciform ligament. Tumor was identified sonography. Inflow control was performed by intermittent Pringle maneuver with extracoporeal tournique. Liver panrechyma transection was performed by CUSA and Harmonica. Tumors were resected en-bloc. Hemovac was placed over right subphrenic. After specimen removal, CRS took for loop ileostomy.
- Estimated blood loss: 1275 c.c
- 器械數量: 正確
- 紗布數量: 正確

Image

- PAINT
  - abdomen

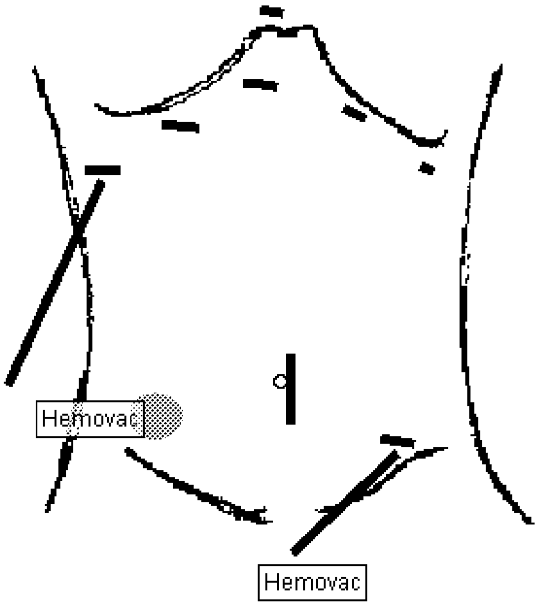

- S6

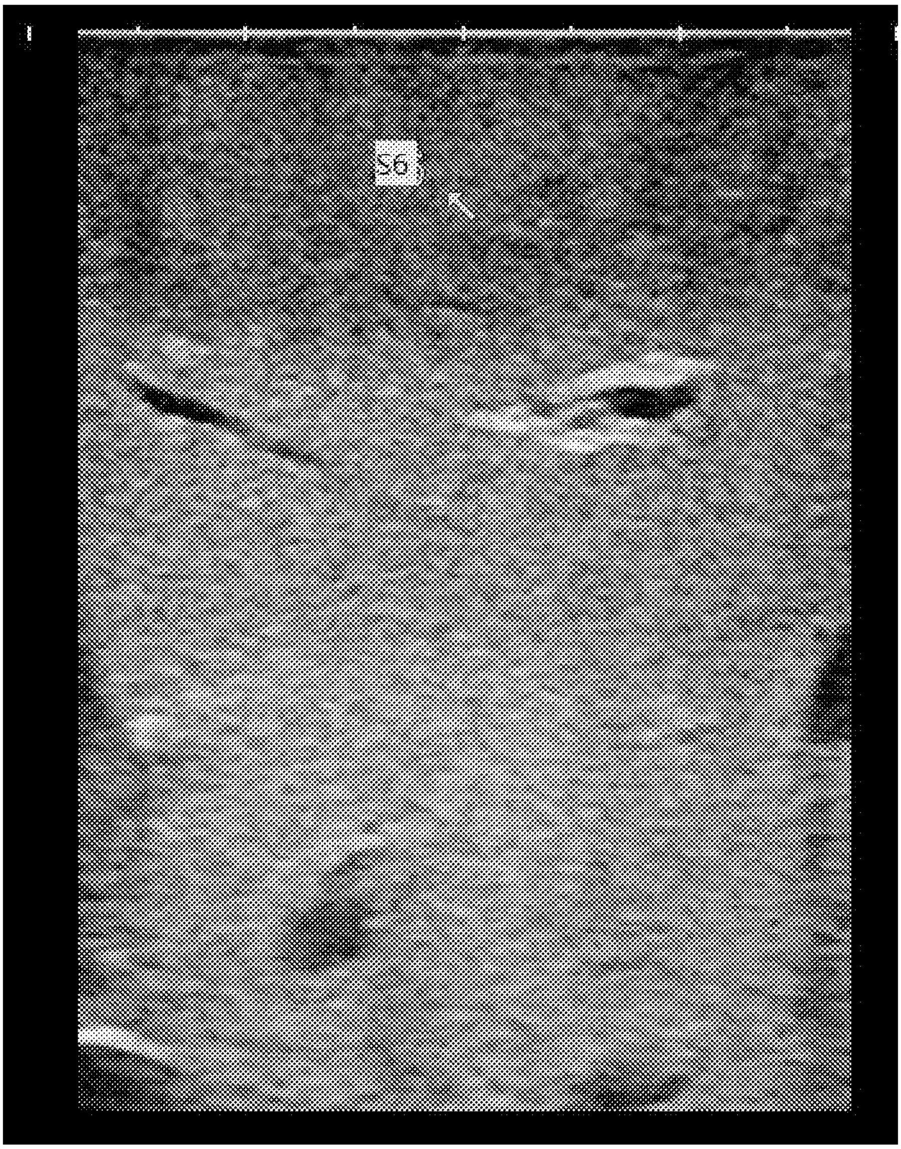

deep S5

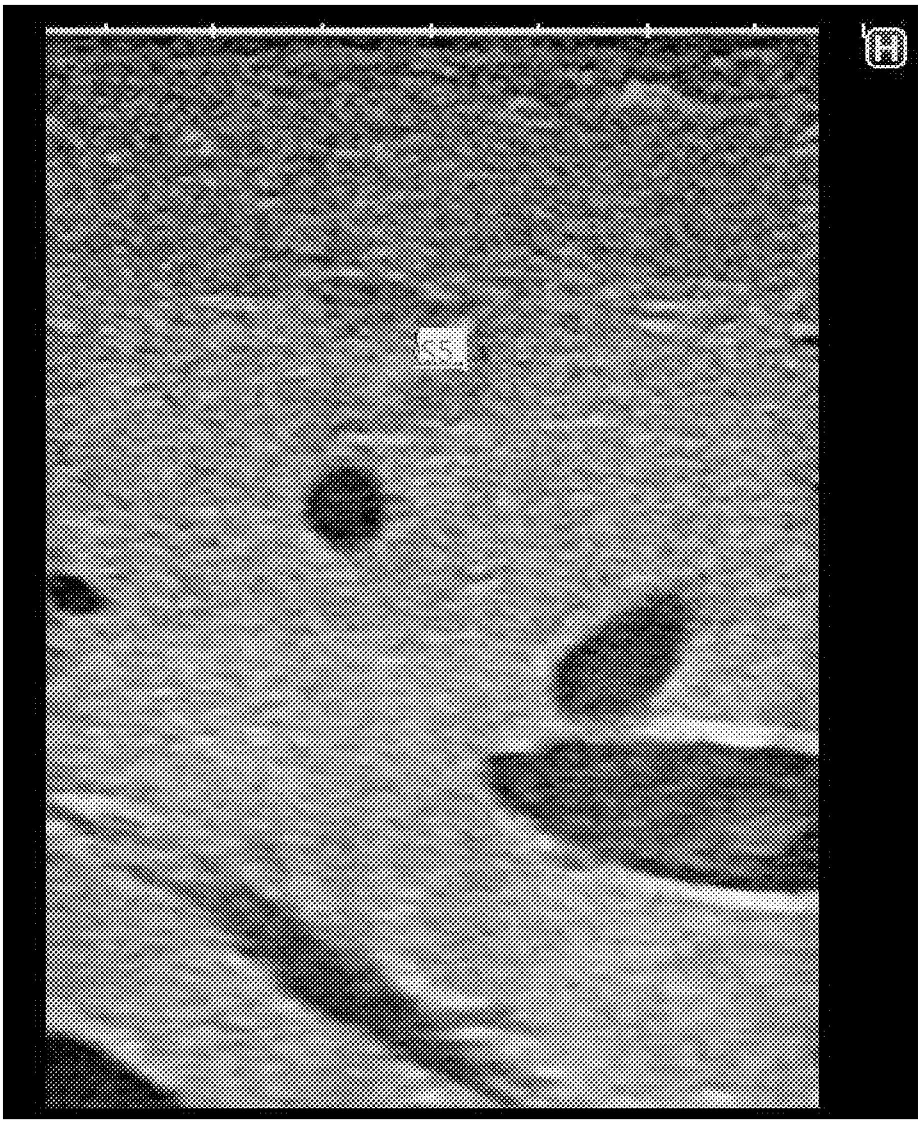

S1 cyst

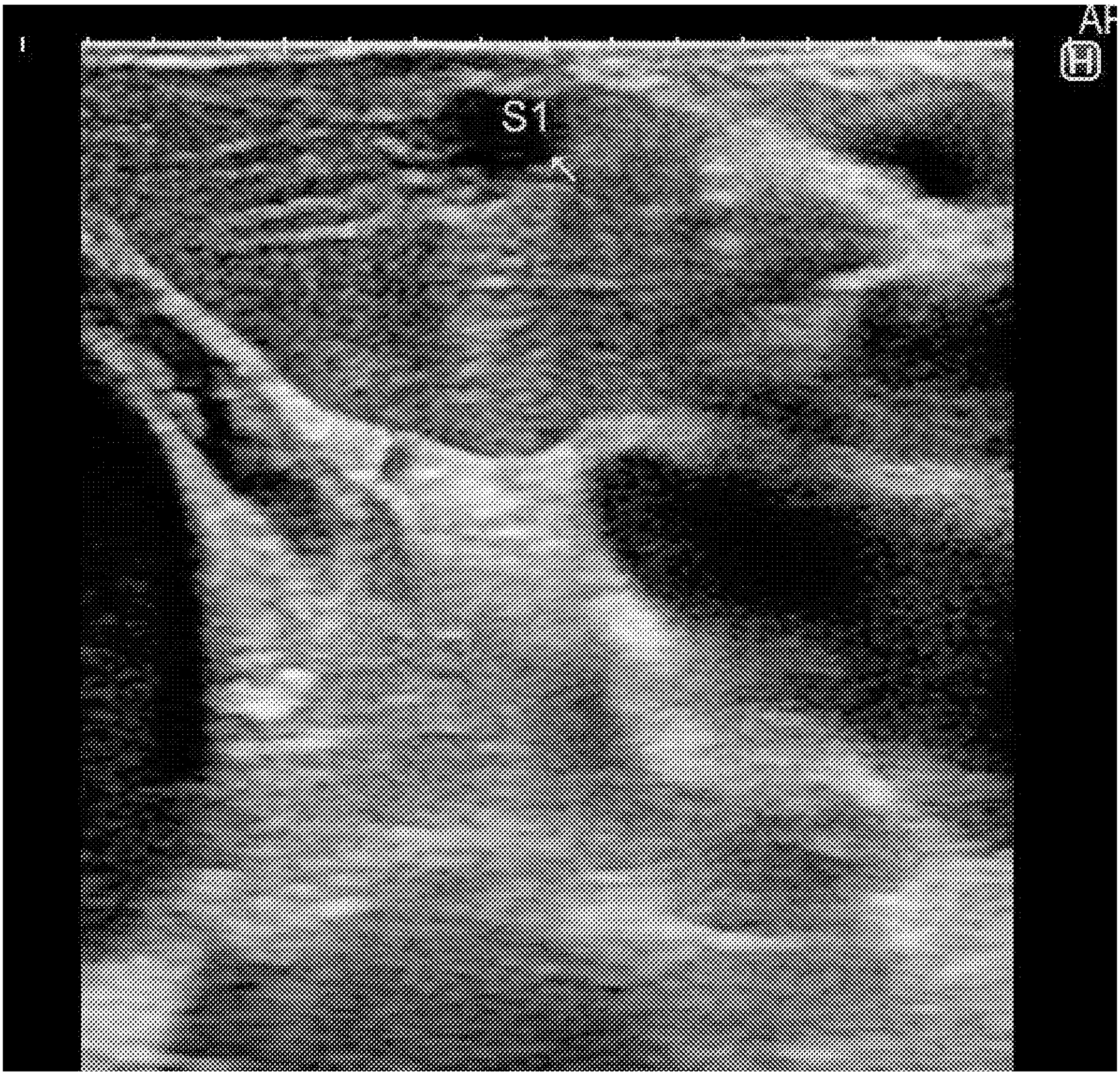

S8 cyst

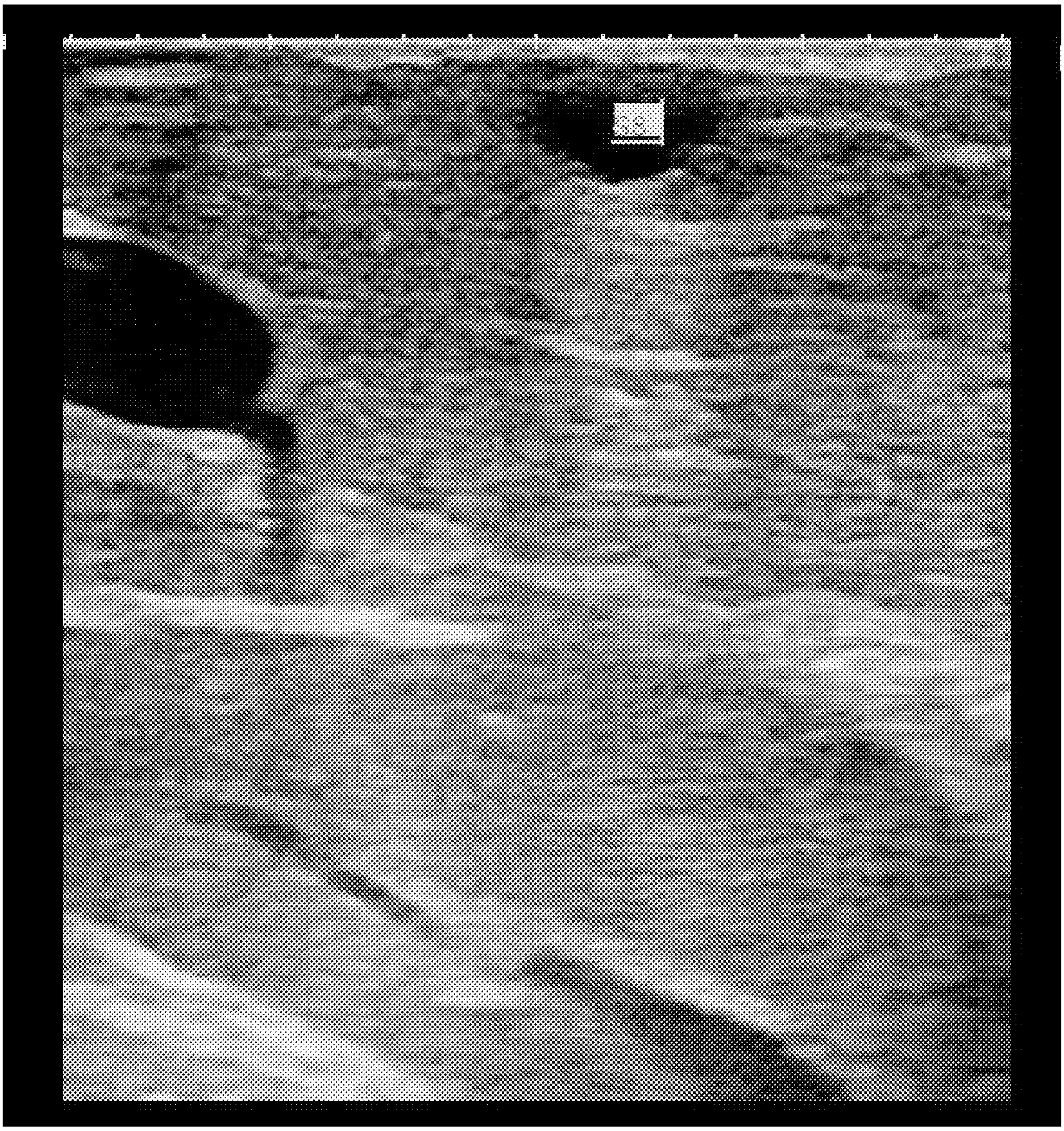

Gallbladder

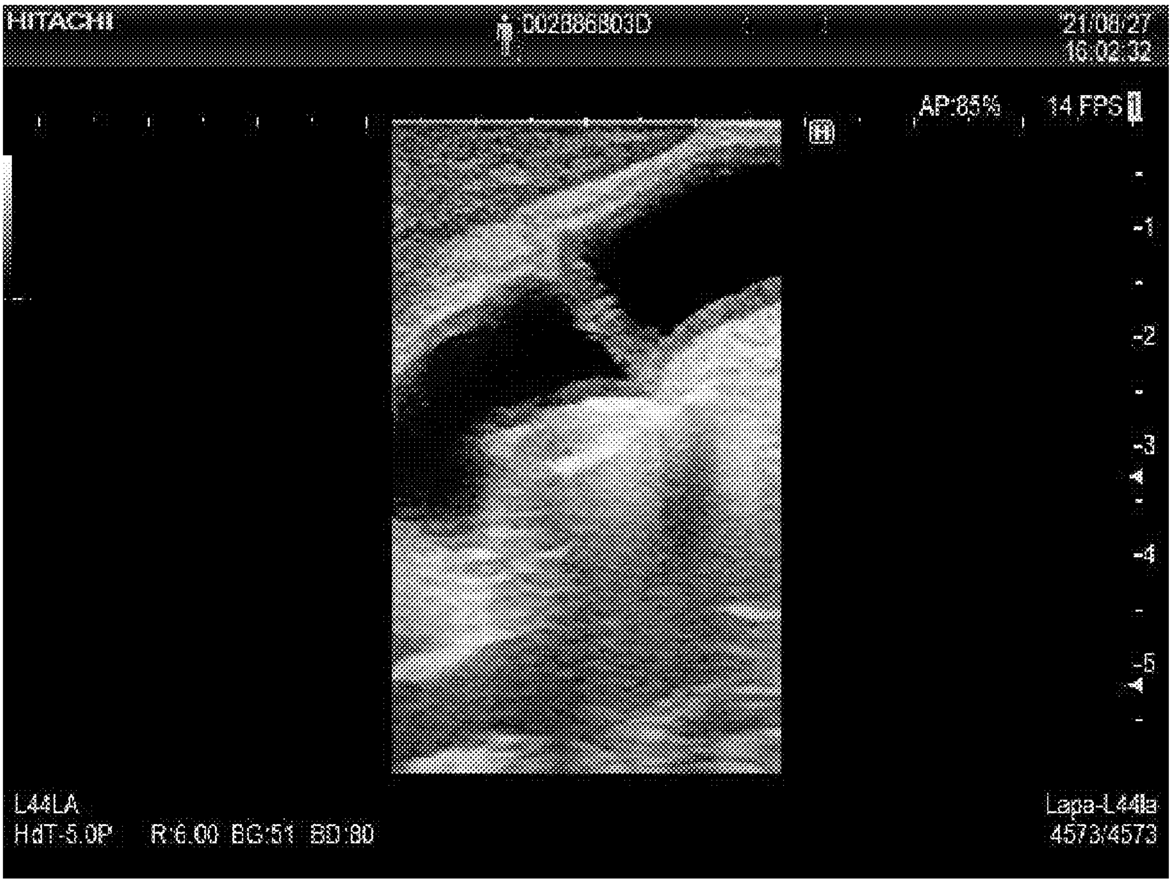

S6

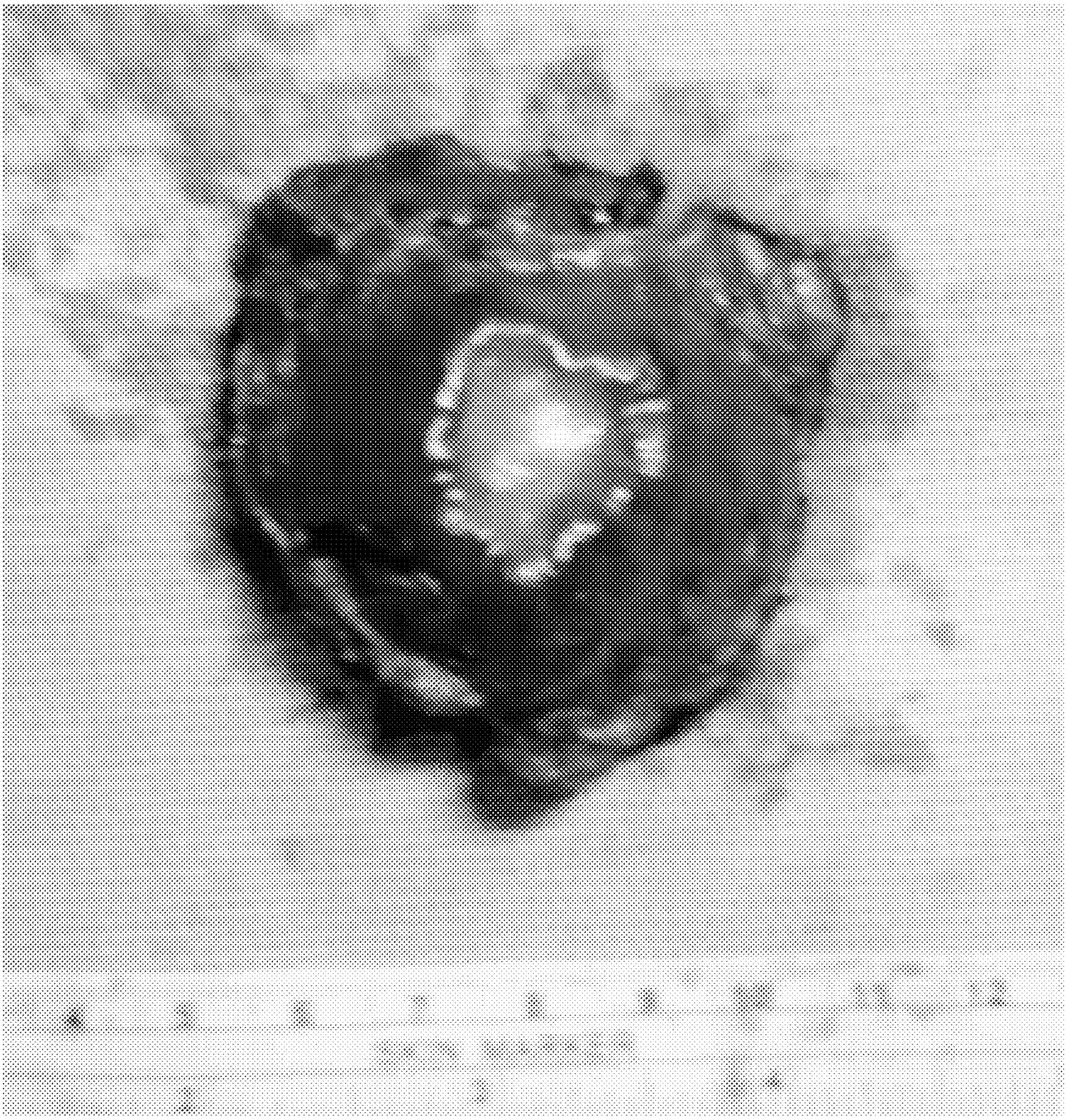

S6

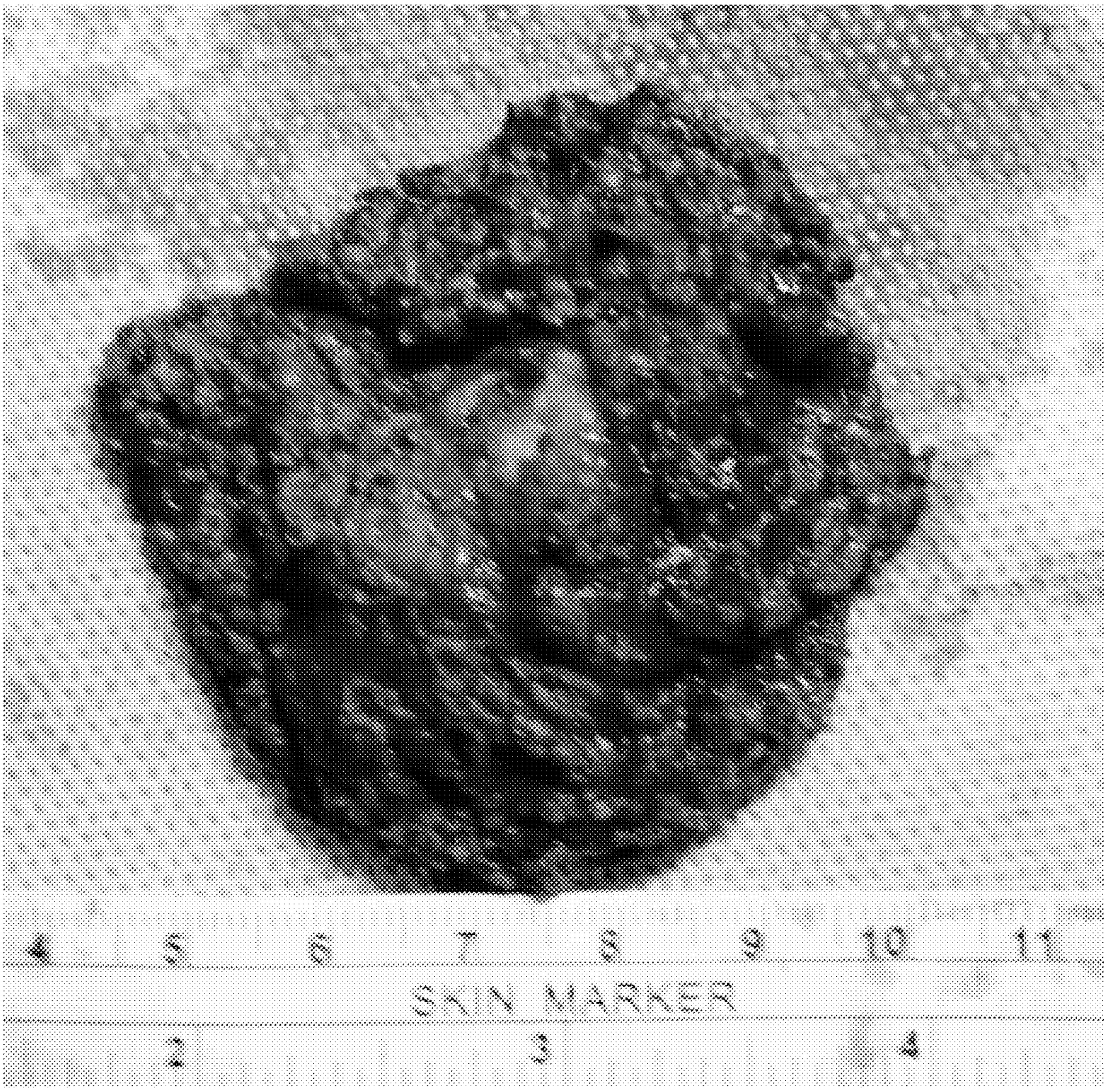
4 5 6 7 8 9 10 11
SKIN MARKER
2 3 4

S6

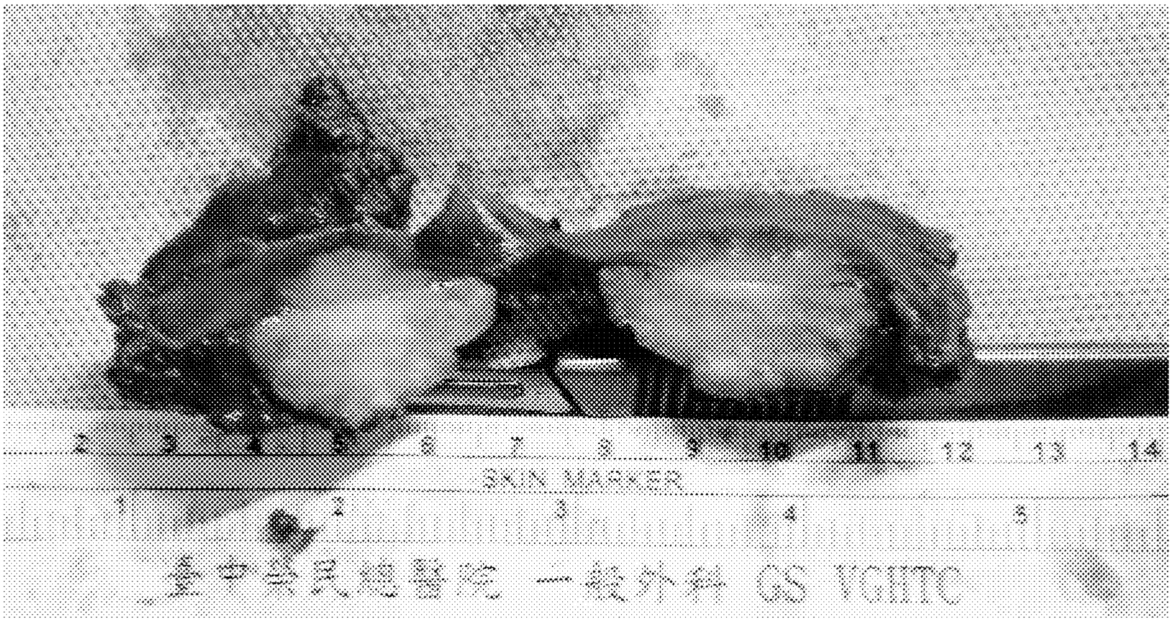
2 3 4 5 6 7 8 9 10 11 12 13 14
SKIN MARKER
1 2 3 4 5
臺中榮民總醫院 一般外科 GS VGHTC

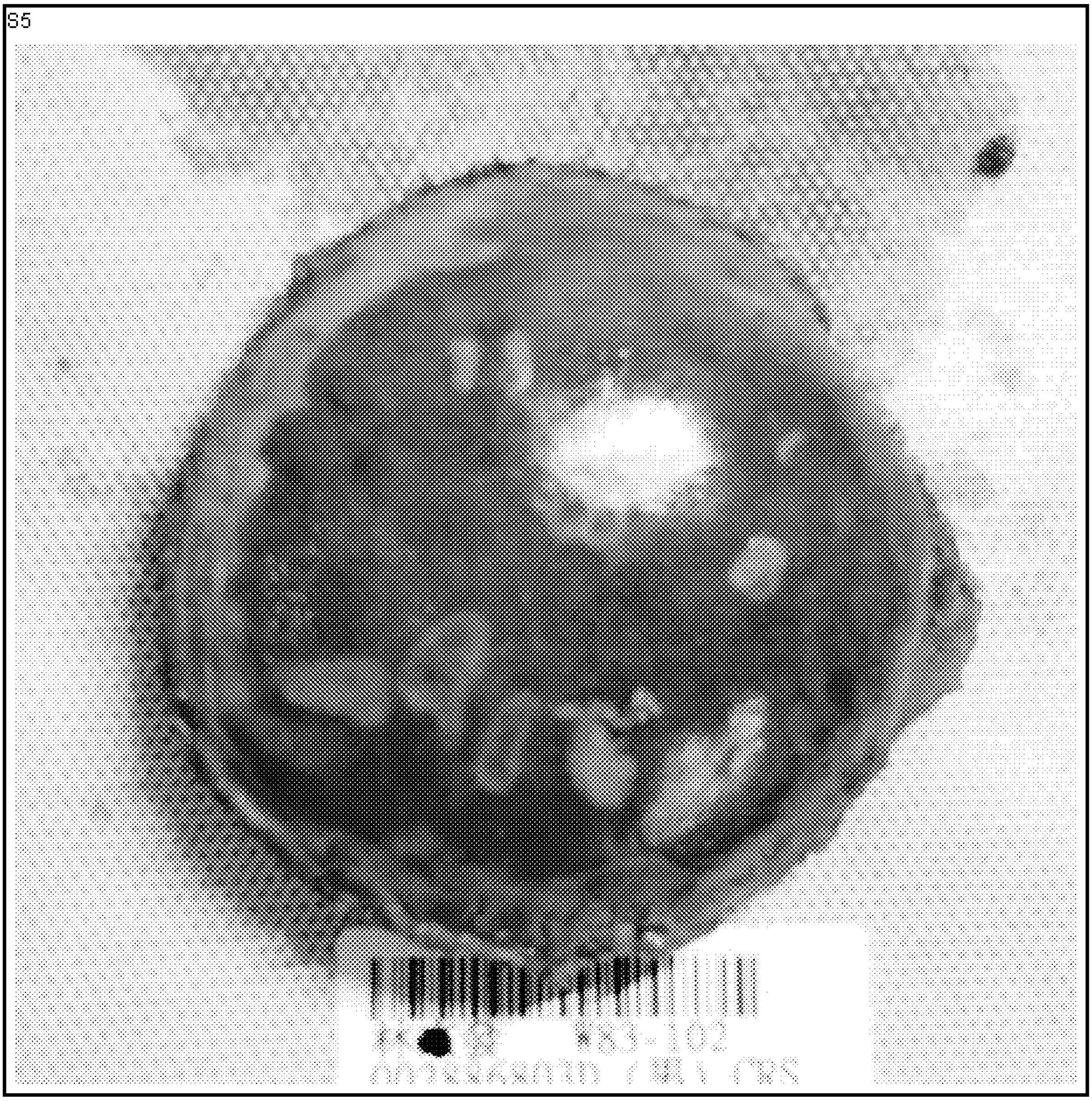
S5

S5

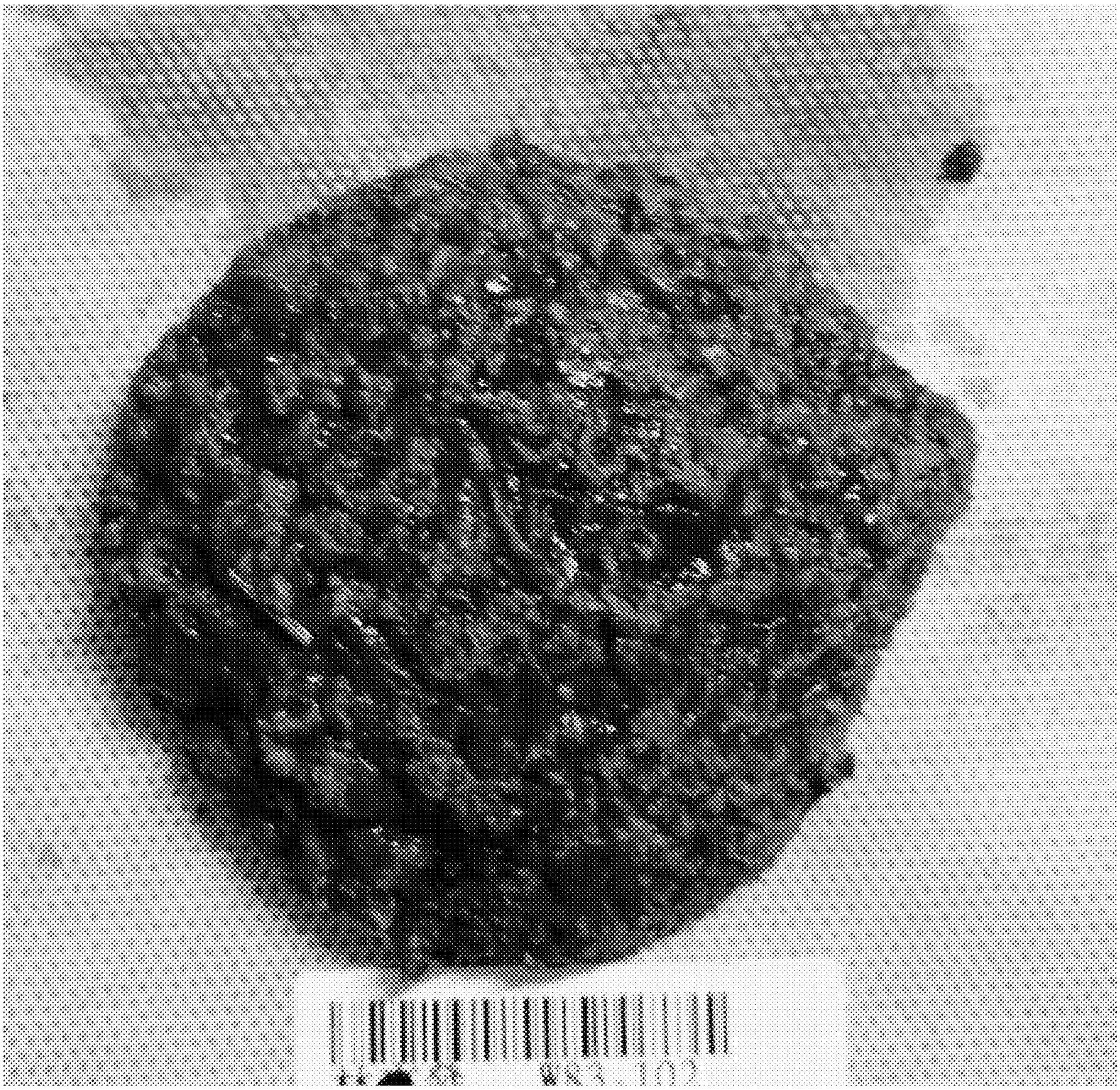

S5

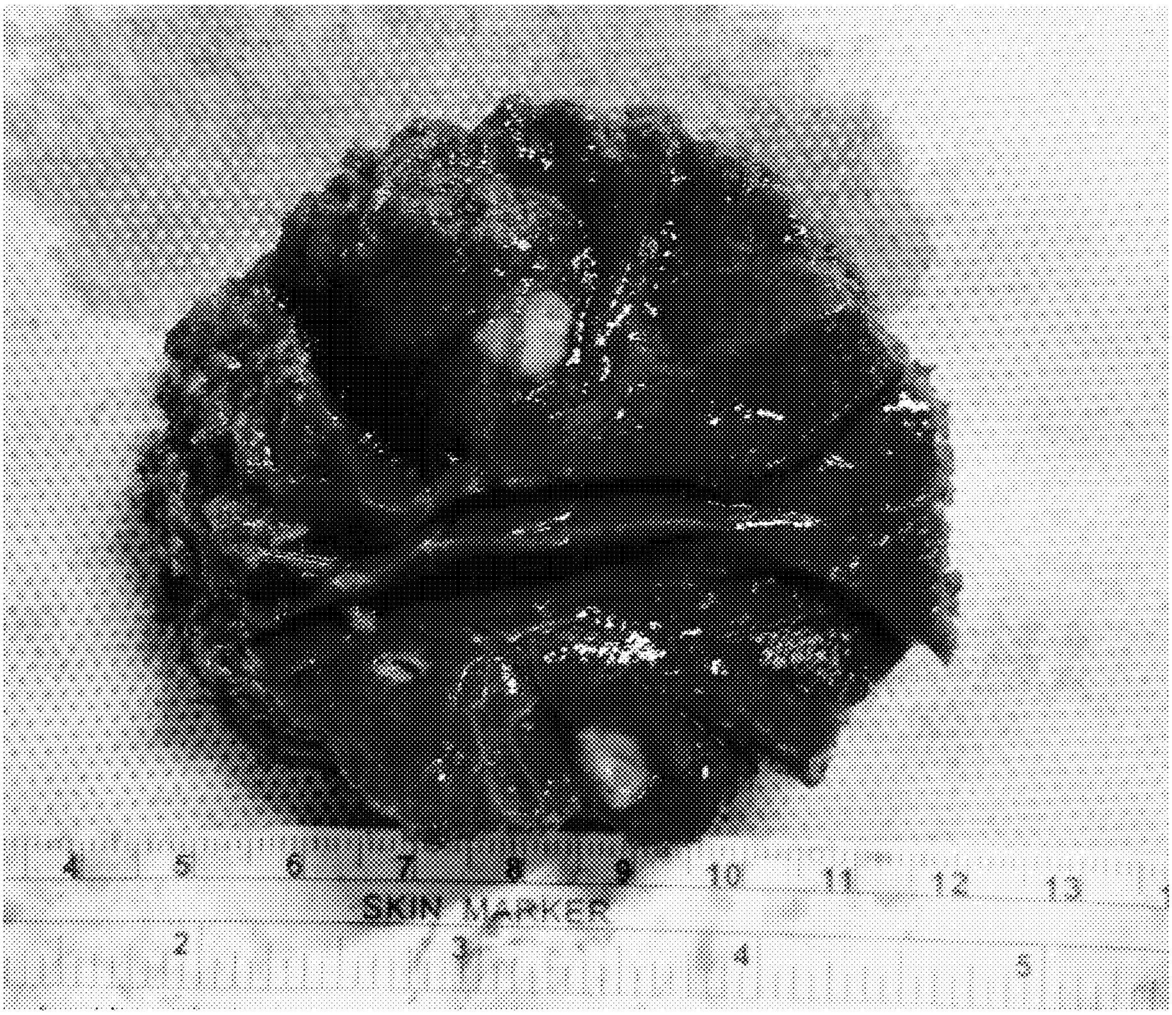

S6

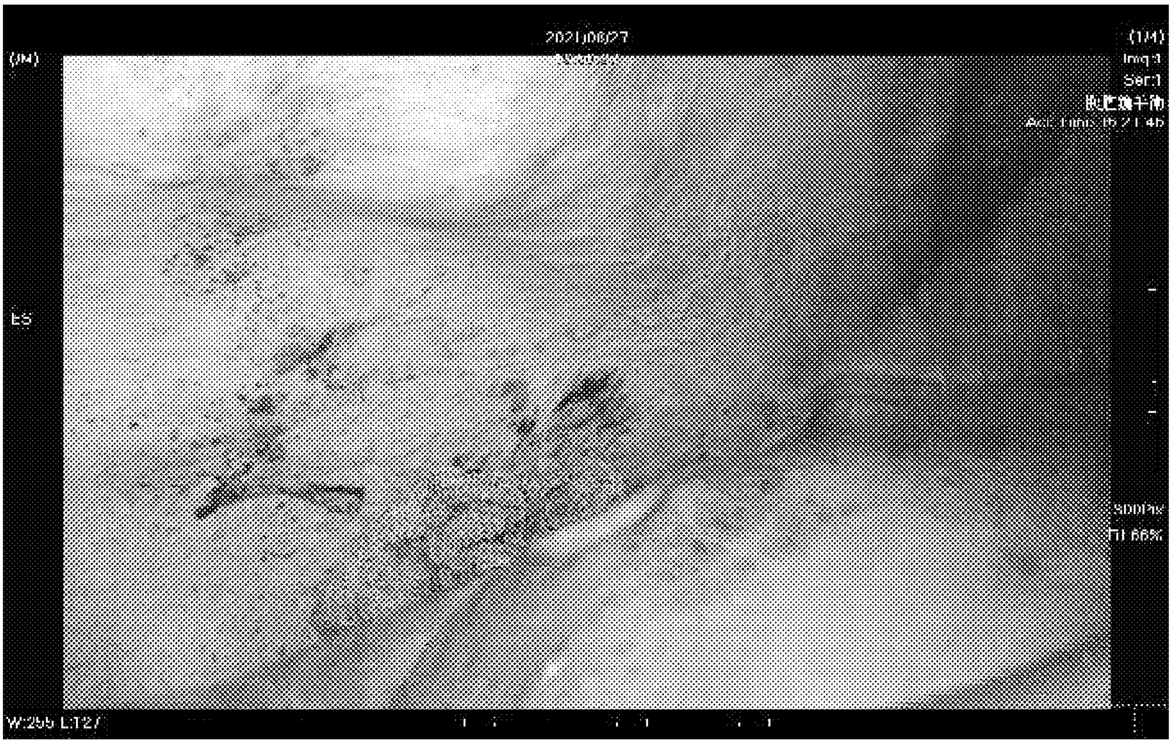

S5 surface

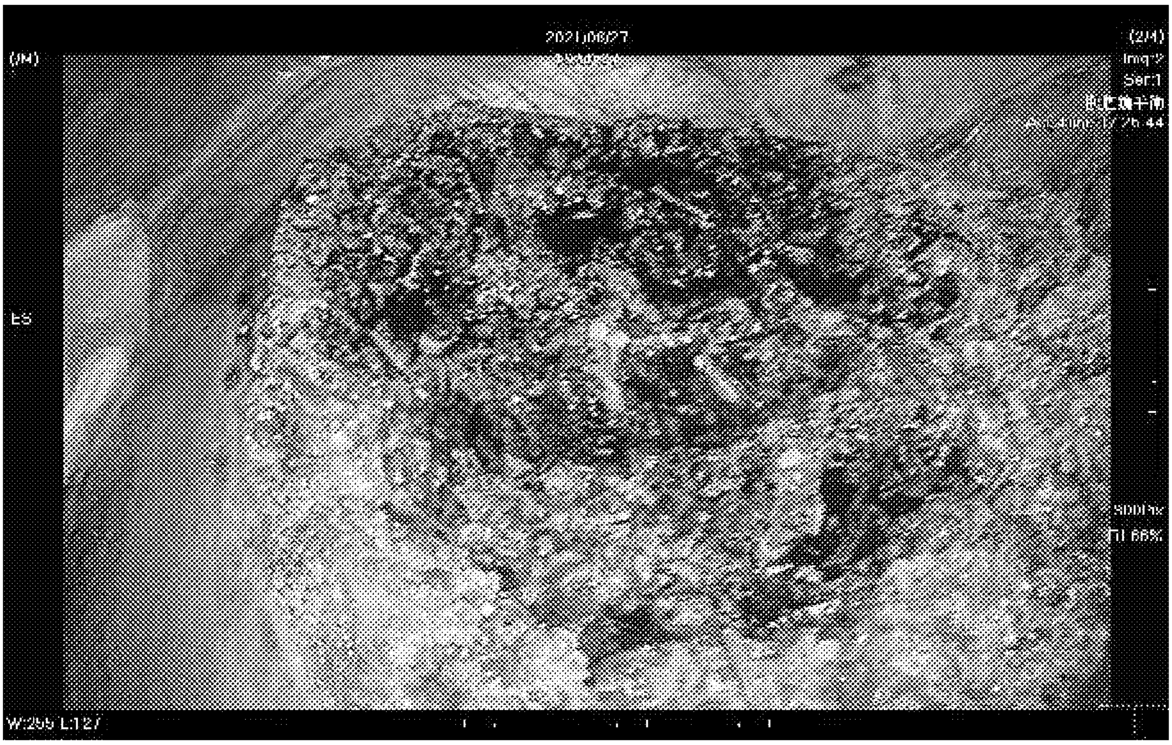

S6 surface

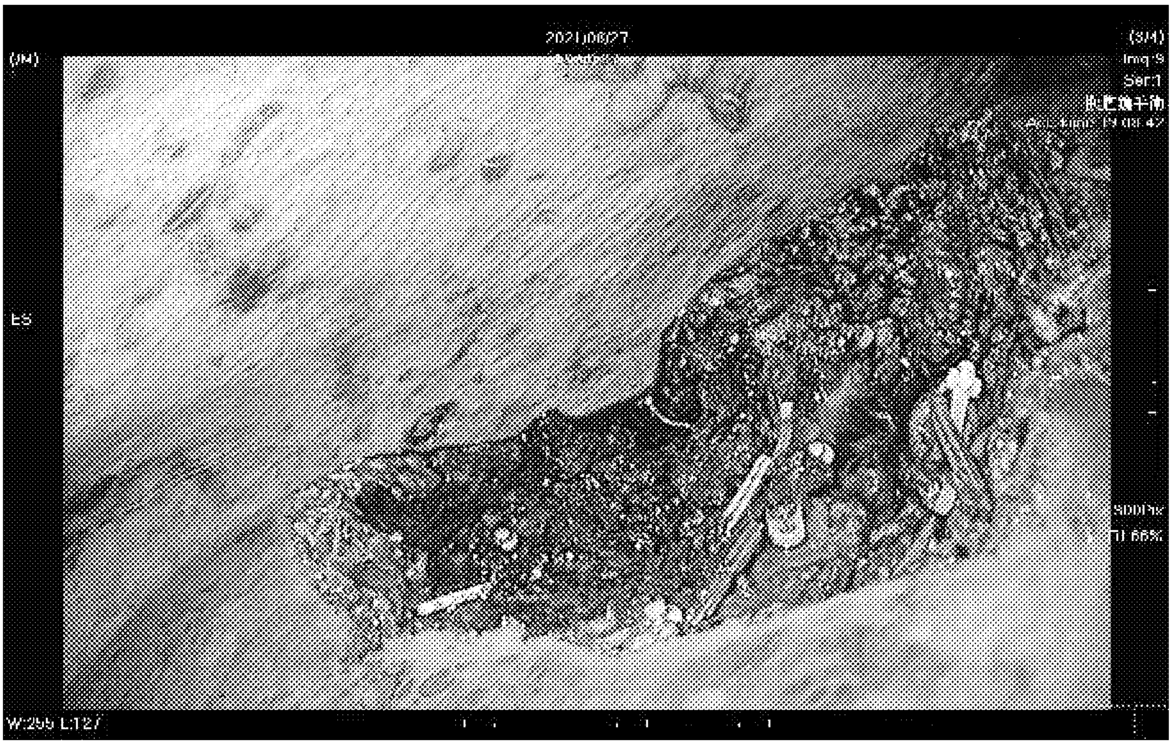

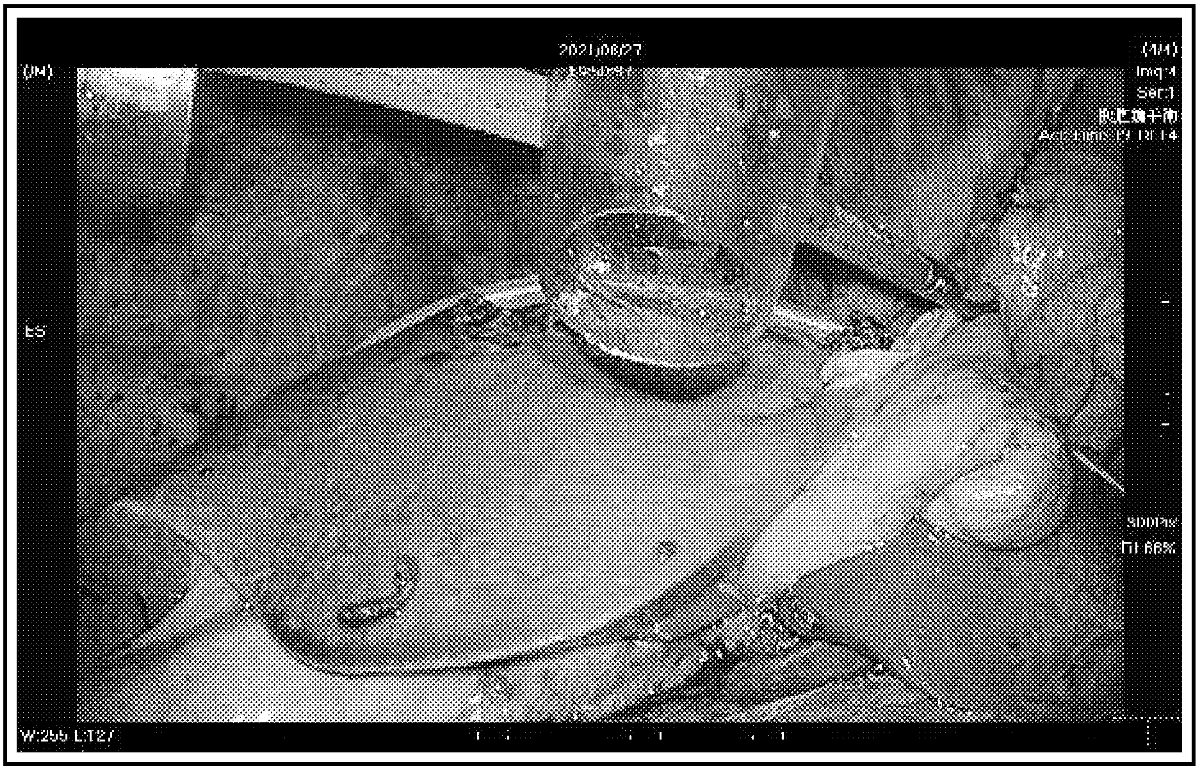

編輯主任 2062F蔣鋒帆、主治 2178L劉嘯天

正式摘要**(電子簽章：000000223723716)**

		請選擇...reporter			

doctorRank:B
